# Identification of new adventitious rooting mutants amongst suppressors of the *Arabidopsis thaliana superroot2* mutation

**DOI:** 10.1093/jxb/eru026

**Published:** 2014-03-04

**Authors:** Daniel Ioan Pacurar, Monica Lacramioara Pacurar, John Desmond Bussell, Joseli Schwambach, Tiberia Ioana Pop, Mariusz Kowalczyk, Laurent Gutierrez, Emilie Cavel, Salma Chaabouni, Karin Ljung, Arthur Germano Fett-Neto, Doru Pamfil, Catherine Bellini

**Affiliations:** ^1^Umeå Plant Science Centre, Department of Plant Physiology, Umeå University, 90187 Umeå, Sweden; ^2^University of Agricultural Sciences and Veterinary Medicine, 400372 Cluj Napoca, Romania; ^3^Umeå Plant Science Centre, Department of Forest Genetics and Plant Physiology, Swedish University of Agricultural Sciences, SE-90183 Umeå, Sweden; ^4^Australian Research Council Centre of Excellence in Plant Energy Biology, University of Western Australia, Crawley WA 6009, Australia; ^5^Centro de Biotecnologia, Laboratório de Fisiologia Vegetal, Universidade Federal do Rio Grande do Sul, 9500, CP15005, CEP 91501–970, Porto Alegre, RS, Brazil; ^6^Université de Picardie Jules Verne, CRRBM & BIOPI EA3900, 80039 Amiens, France; ^7^Institut Jean-Pierre Bourgin, UMR1318 INRA-AgroParisTech 78026 Versailles Cedex, France

**Keywords:** Adventitious roots, *Arabidopsis*, auxin, ethylene, *superroot*, suppressors.

## Abstract

The plant hormone auxin plays a central role in adventitious rooting and is routinely used with many economically important, vegetatively propagated plant species to promote adventitious root initiation and development on cuttings. Nevertheless the molecular mechanisms through which it acts are only starting to emerge. The *Arabidopsis superroot2-1* (*sur2-1*) mutant overproduces auxin and, as a consequence, develops excessive adventitious roots in the hypocotyl. In order to increase the knowledge of adventitious rooting and of auxin signalling pathways and crosstalk, this study performed a screen for suppressors of *superroot2-1* phenotype. These suppressors provide a new resource for discovery of genetic players involved in auxin signalling pathways or at the crosstalk of auxin and other hormones or environmental signals. This study reports the identification and characterization of 26 *sur2-1* suppressor mutants, several of which were identified as mutations in candidate genes involved in either auxin biosynthesis or signalling. In addition to confirming the role of auxin as a central regulator of adventitious rooting, *superroot2* suppressors indicated possible crosstalk with ethylene signalling in this process.

## Summary

Auxin is a major regulator of adventitious rooting and, to better understand its role, we identified suppressor mutants of *superroot2-1.* This provides new resources for the discovery of genetic players involved in auxin signalling or auxin crosstalk with other hormones.

## Introduction

Adventitious root (AR) formation defines the process whereby roots develop at any location other than from a root. In horticulture and forestry in which asexual plant propagation is widely used, AR formation from cuttings is a necessary step. Adventitious rooting is a complex organogenic process controlled by multiple endogenous and environmental factors (Geiss *et al.*, 2009; da Costa *et al.*, 2013), among which the plant hormone auxin plays a central role. Auxin has long been known as the rooting hormone (Haissig and Davis, 1994) and is used to promote development of AR in stem cuttings, but other phytohormones can also promote or accelerate rooting and are often used in association with auxin treatments (Kevers *et al.*, 2009). In *Arabidopsis*, mutants overproducing auxin, such as the *superroot* mutants *sur1* and *sur2* or the *yucca* mutants, spontaneously develop AR in the hypocotyl when grown under light (Boerjan *et al.*, 1995; Delarue *et al.*, 1998; Zhao *et al.*, 2001). Although auxin plays a central role in the determination of rooting capacity, its use is not always effective in promoting rooting of recalcitrant genotypes (Fogaça and Fett-Neto, 2005), highlighting the fact that in this developmental process, as in others, auxin interacts either with other endogenous factors or environmental stimuli such as light (Fett-Neto *et al.*, 2001; Sorin *et al.*, 2005).

Besides auxin, ethylene can also promote adventitious rooting. The ethylene-insensitive never ripe tomato mutant developed fewer AR than the wild type (Clark *et al.*, 1999). More recently it was demonstrated that the positive role of ethylene in AR formation is likely due to a modulation of auxin transport, which is a central point of ethylene–auxin crosstalk (Negi *et al.*, 2010). Ethylene could also promote rooting by stimulating cytokinin catabolism (Kuroha and Sakakibara, 2007). Cytokinin has been shown to act as a rooting inhibitor (Geiss *et al.*, 2009; Ramirez-Carvajal *et al.*, 2009). Therefore, downregulation of the endogenous cytokinin content via catabolism or repression of the cytokinin signalling pathway promotes adventitious rooting. Abscisic acid (ABA), like cytokinins, appears to be a negative regulator of adventitious rooting. The ABA-deficient tomato mutants *flacca* and *notabilis* produce an excess of AR on the stems (Tal, 1966). The AR phenotype of the *notabilis* mutant could be restored to wild-type phenotype by expressing *SpNCED1*, involved in ABA biosynthesis (Thompson *et al.*, 2004). Niu *et al.* (2013) recently demonstrated that a high concentration of endogenous gibberellins in the stem of tobacco plants had an inhibitory effect on the early steps of adventitious root initiation in tobacco cuttings. In addition, it was shown that adventitious rooting is regulated by the stress-related hormone jasmonic acid (JA; Ahkami *et al.*, 2009; Fattorini *et al.*, 2009; Gutierrez *et al.*, 2012). Strigolactones, a new class of phytohormones, were recently shown to have an inhibitory effect on adventitious rooting both in *Arabidopsis* and pea (Rasmussen *et al.*, 2012). They might act by modulating the auxin level in the cells or tissues from which AR originate. Among endogenous compounds, it has also been reported that alkamides and nitric oxide (Pagnussat *et al.*, 2003; Pagnussat *et al.*, 2004; Campos-Cuevas *et al.*, 2008; Mendez-Bravo *et al.*, 2010), polyamines (Rey *et al.*, 1994; Heloir *et al.*, 1996), and flavonoids (Curir *et al.*, 1990) are important players in the regulation of adventitious rooting.

Although these studies clearly indicate the importance of hormone homeostasis and hormone crosstalk, they do not reveal specific molecular mechanisms and additional investigation is needed to better understand the mechanism of auxin in the control of AR formation. In rice (*Oryza sativa*), *CROWN ROOTLESS1* (*CRL1*)/*ADVENTITIOUS ROOTLESS1* (*ARL1*) (Inukai *et al.*, 2005; Liu *et al.*, 2005) encodes a member of the plant-specific ASYMMETRIC LEAVES2/LATERAL ORGAN BOUNDARIES (LOB) protein family and has been shown to control AR development. Disruption of *CRL1*/*ARL1* prevents initiation of adventitious crown root primordia in rice. *CRL1*/*ARL1* is an auxin-responsive gene that contains two putative auxin-response elements (AREs) in its promoter region. Its induction by auxin requires the degradation of Aux/IAA proteins and it was shown that the proximal AREs specifically interacted with a rice *AUXIN RESPONSE FACTOR* (*ARF*) and acted as a *cis*-motif for *CRL1* expression (Inukai *et al.*, 2005). *CRL1*/*ARL1* can be considered as a positive regulator for crown root formation in rice. Similarly, the current study group has shown that AR initiation in *Arabidopsis* hypocotyls is controlled by a subtle balance between the negative regulator *ARF17* and the positive regulators *ARF6* and *ARF8*, which display overlapping expression domains, interact genetically, and regulate each other’s expression at both transcriptional and post-transcriptional levels (Gutierrez *et al.*, 2009). More recently, this study group has shown that, in contrast to *ARF17*, *ARF6* and *ARF8* are positive regulators of the auxin-inducible genes *GH3-3*, *GH3-5*, and *GH3-6* that fine-tune AR initiation in *Arabidopsis* hypocotyls by modulating JA homeostasis (Gutierrez *et al.*, 2012). These findings highlight a regulatory module at the crosstalk between jasmonate and auxin signalling pathways.

Genetic screens for suppressors have often been used to further investigate gene functions and to dissect signal transduction pathways. In *Arabidopsis*, suppressor screens have been used to identify genes/mutants that function in, for example, the auxin (Cernac *et al.*, 1997), gibberellin (Jacobsen and Olszewski, 1993; Wilson and Somerville, 1995), and abscisic acid (Koorneef *et al.*, 1982) pathways. The mutation *sur2-1* causes a loss of function of the cytochrome P450 *CYP83B1*, and the mutant spontaneously produces an excess of AR (Delarue *et al.*, 1998).

In order to identify more genes that might control the initiation or development of AR, the current work used the *sur2-1* AR trait and performed a screen for suppressor mutants that produced fewer AR than *sur2-1*. This work isolated 46 such mutants representing 34 groups of complementation. This report describes mapping of 32 of these mutants and the phenotypic characterization of independent *sur2-1* suppressors covering 26 complementation groups.

## Materials and methods

### Identification of *sur2-1* suppressors

In order to avoid selecting wild-type seedlings due to potential contamination of the mutagenized population with wild-type seeds, the *glabra1* mutation was introgressed in the *sur2-1* mutant background. The *glabra1* mutant was identified in the Versailles collection of T-DNA insertion lines and was therefore in the same genetic background as *sur2-1*. Homozygote seeds from the double-mutant *superroot2-1gl1* (*sur2-1gl1*) (ecotype Wassilewskija, Ws-4) were mutagenized with ethyl methanesulphonate, as described in Santoni *et al.* (1994). The M2 progeny of 2345 independent lines was collected and screened as follows: seeds were surface sterilized, sown *in vitro*, stratified for 48h at 4 °C, then transferred to a plant growth chamber but kept in the dark until the hypocotyls reached sizes of 5–6mm. They were then transferred to the light for 7 d, as described previously (Sorin *et al.*, 2005). The conditions in the controlled-environment chambers were as follows: 130 μE m^–2^ s^–1^ irradiance on average, 16/8 light/dark cycle, 22/15 °C. 60% relative humidity. For dark growth conditions, Petri dishes were wrapped with three layers of aluminium foil and placed vertically. After 7 d in the light, the AR were counted on the hypocotyls. Seedlings that produced fewer AR than *sur2-1gl1* were transferred to soil and grown to set seeds. The M3 was then rescreened to confirm the suppressor phenotype in the progeny. A schematic of the screening procedure is presented on Supplementary Fig. S1 available at *JXB* online.

### Genetic segregation and complementation analysis

The suppressors were backcrossed twice with the original *sur2-1gl1* line used as the female parent, except for the male sterile suppressor *677*/*2191*, where *sur2-1gl1* was used as male parent. Segregation analysis was performed in the progeny of the second backcross (Supplementary Table S1). Suppressor mutants showing similar phenotypes that were identified early in the screening process were crossed to each other for complementation analysis. Once the coarse mapping information was available, only those mutants that were mapped in the same region were crossed for complementation analyses.

### Mapping of *sur2-1* suppressors

To map the suppressor mutations (Ws-4 background), phenotyped mutant seedlings that yielded fewer AR than *sur2-1gl1* were identified in an F2 population obtained by crossing the suppressor mutant with *atr4-1*, an allele of the *sur2* mutant in a Columbia-0 (Col-0) background (Smolen and Bender, 2002). Using standard protocols, genomic DNA was extracted from entire mutant seedlings grown *in vitro* as previously described by (Sorin *et al.*, 2005) and used as template for mapping as described by Pacurar *et al.* (2012).

### Hypocotyl and root measurements

Hypocotyl and root measurements were performed as described by Gendreau *et al.* (1997). Seedlings were grown vertically in Petri dishes. The plates were placed directly in the light (without prior etiolation) for 7 d. Plates were photographed and hypocotyls and roots measured using ImageJ software (http://rsb.info.nih.gov/ij/index.html). All measurements were performed on three independent biological replicates with a minimum of 40 seedlings each.

### Counting of adventitious and lateral root number

ARs were counted on hypocotyls of etiolated seedlings, following the same protocol described above for the screening. Lateral roots were counted on light grown seedlings used for the hypocotyl and root measurements. All counting was performed on three independent biological replicates with a minimum of 40 seedlings each.

### Determination of free IAA concentrations

The screen and characterization of the suppressor mutants was performed over a long period of time. Therefore, the endogenous free IAA content was measured in two periods (2008 and 2011) using different methods. Seedlings were grown *in vitro*, in controlled diurnal environment with a 16/8 light/dark cycle (average irradiance 130 μE m^–2^ s^–1^) and 60% relative humidity. The root was removed and the aerial parts were pooled to obtain an average of 15mg fresh weight per sample. Samples were extracted, purified and analysed by liquid chromatography multiple-reaction monitoring mass spectrometry (LC-MRM-MS), as described by Kowalczyk and Sandberg (2001) or by gas chromatography selected-reaction monitoring mass spectrometry (GC-MRM-MS) as described by Andersen *et al.* (2008). In the two experiments, measurements were performed with three independent biological replicates.

### RNA isolation and cDNA synthesis

RNAs from wild type and suppressor mutants were prepared as previously described (Gutierrez *et al.*, 2009). RNA (5 μg) were treated with DNaseI using a DNAfree Kit (Ambion) and cDNA was synthesized as described by Gutierrez *et al.* (2012). All cDNA samples were tested by PCR using specific primers flanking an intron sequence to confirm the absence of genomic DNA contamination.

### Real-time PCR experiments and data analysis

Transcript abundance was assessed by quantitative real-time (RT) PCR according to this study group’s previously described procedure (Gutierrez *et al.*, 2012). All quantifications were repeated with three independent biological replicates.

Steady-state levels of uncleaved *ARF* transcripts were quantified using primers spanning the miRNA target site. The following standard protocol was applied for the amplification of each mRNA: 10min at 95 °C, followed by 40 cycles at 95 °C for 10 s, 60 °C for 15 s (except for *GH3.5* for which the annealing temperature was 65 °C), and 72 °C for 15 s. The sequences of primers used for all target genes are presented in Supplementary Table S2.

*APT1* and *TIP41* had previously been validated as the most stably expressed genes of the 11 tested (Gutierrez *et al.*, 2009) and were used to normalize the RT-PCR data. The normalized expression patterns obtained using both reference genes were similar, so only the data normalized with *APT1* are shown. CT and E values were used to calculate expression using the formula E_T_^(CT^_Cal_^–CT^_M_^)^/E_R_^(CT^_Cal_^–CT^_M_^)^ where Cal and M are subscripts to the superscript CT. where T is the target gene and R the reference gene, CT is the crossing threshold value, M refers to cDNA from the mutant line, and Cal refers to cDNA from the calibrator. The data for the suppressor mutants are presented as relative to the calibrator (i.e. the wild type or *sur2-1gl1*). All RT-PCR results presented are means from three independent biological replicates. For each independent biological replicate, the relative transcript amount was calculated as the mean of three technical replicates, using the method for calculation of standard errors in relative quantification recommended by Rieu and Powers (2009).

### Statistical analysis

Statistical analysis was performed using the GraphPad Prism version 6.0d for Mac (www.graphpad.com). One-way ANOVA combined with the Tukey’s multiple comparison post-test was used for multiple means’ comparisons. Linear regression and Pearson correlation coefficient *r* were calculated using the GraphPad package.

## Results and discussion

### Suppressor mutant isolation and genetic characterization

A total of 2345 individually harvested M2 seed stocks derived from ethyl methanesulphonate-mutagenized *sur2-1gl1* double-mutant plants were screened for a phenotype showing fewer adventitious roots than *sur2-1gl1.* In order to limit finding mutants altered in the expression of genes already known to be involved in the control of root development and/or auxin signalling, suppressor mutants that had a shorter primary root or fewer or no lateral roots were eliminated as much as possible.

A total of 46 mutants showing fewer AR than *sur2-1gl1* double mutants were identified, and the suppressor phenotype was confirmed in the M3 generation and after at least two backcrosses with *sur2-1gl1*, as described in Supplementary Fig. S1. Segregation analysis was performed for 33 of these mutants (Supplementary Table S1). Of the analysed mutants, all but one (no. 1626) were recessive. In some cases, the proportion of plants exhibiting the suppressor phenotype was lower (*P*<0.05) than the expected 3:1 *sur2-1gl1*:mutant segregation (Supplementary Table S1). No embryo lethality or germination problems were observed, suggesting that these suppressors could be double mutants. In two cases (nos. 266 and 2037), several alleles were identified ruling out this hypothesis, at least for these two mutants.

**Fig. 1.**
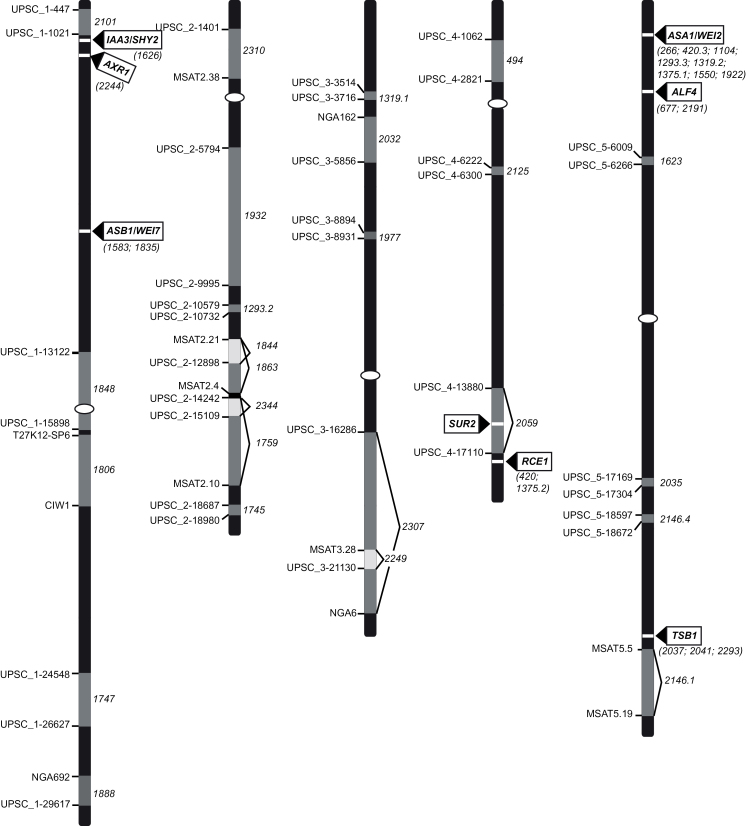
Chromosomal localization of *sur2-1* suppressor mutations. From left to right: chromosomes 1, 2, 3, 4, and 5. Black indicates chromosomes; horizontal white bars indicate the positions of identified suppressor genes, whose names are indicated in boxes with arrows; numbers in brackets indicate alleles. Light and dark grey boxes indicate regions containing the mutations (numbers on the right of the chromosomes) that still need to be identified, each flanked by two indel markers (identified on the left of the chromosomes).

**Fig. 2.**
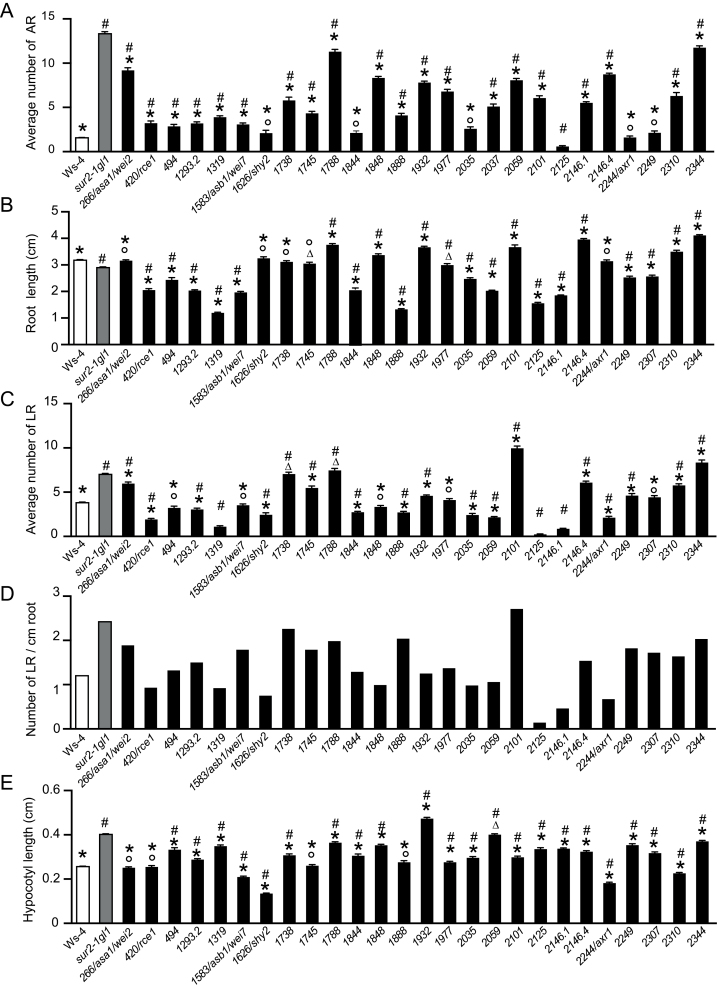
Characterization of representative suppressor seedlings. (A) Adventitious roots were counted on seedlings of the wild-type Ws-4, *sur2-1gl1* mutant and suppressor mutant lines; seedlings were first etiolated in the dark, until their hypocotyls were 6mm long, then transferred to the light for 7 d. (B) Root length was measured on seedlings grown *in vitro* directly in light conditions for 7 d and averaged as described in Materials and methods. (C) Numbers of emergent lateral roots were counted on the same seedling as in B and averaged. (D) Lateral root density was estimated by dividing the average number of lateral roots (C) by the average root length (B). (E) Hypocotyl length was measured on the same seedlings as in B. For A-E, at least 40 seedlings of each line were analysed and the experiments were repeated three times and the data pooled. Error bars indicate standard error; one-way ANOVA combined with the Tukey’s multiple-comparison post-test was used to compare suppressor lines with wild type and *sur2-1gl1*; asterisks indicate values significantly different from *sur2-1gl1* mutant values (*P*<0.01; *n*>120); hashes indicate values significantly different from wild-type values (*P*<0.01; *n*>120); ○ and Δ indicate values not significantly different from wild type or *sur2-1gl1* values, respectively.

Considering the number of mutants, in the first instance only those that looked alike were crossed to determine complementation groups. In parallel, the mutations were mapped on the chromosomes using the strategy and the InDel markers reported previously (Pacurar *et al.*, 2012). Mutants that were located in the same region were crossed to check for potential allelism. The 46 mutants represented 34 groups of complementation (Supplementary Table S1). Most mutants were single-allele mutants. One particular gene (no. 266) showed a very high mutation rate with eight alleles, while others had two (nos. 420, 677, and 1583) or three (no. 2037) alleles (Fig. 1). These results indicate that, although a rather small number of mutagenized lines were analysed, saturation of the genome was likely reached. Surprisingly, no linkage with any of the markers tested could be found for two mutations (nos. 1738 and 1788). This cannot be explained by a segregation defect since they both clearly segregated as single-locus recessive mutations (Supplementary Table S1) and the phenotype (although mild for no. 1788; Fig. 2A) was easily detectable in the mapping population. The difficulty in positioning these mutations suggests that they might be located in regions with hot spots of recombination.

### Identification of the mutated genes

Several candidate genes were identified based on coarse mapping and similarity of the mutant phenotypes to previously described mutants. These candidate genes were sequenced and eight allelic mutants (nos. 266, 420.2, 1104, 1293.3, 1319.2, 1375.1, 1550, 1922) were readily identified in *ANTHRANILATE SYNTHASE ALPHA 1* / *WEAK ETHYLENE INSENSITIVE 2* (*ASA1*/*WEI2*), two allelic mutants (nos. 1583, 1835) in the *ANTHRANILATE SYNTHASE β 1*/*WEAK ETHYLENE INSENSITIVE 7* (*ASB1*/*WEI7*), and three (nos. 2037, 2041, 2293) in the *TRYPTOPHAN SYNTHASE BETA 1* (*TSB1*) (Fig. 1, Supplementary Fig. S2). These three genes are part of the tryptophan biosynthesis pathway (Last *et al.*, 1991; Niyogi and Fink, 1992; Niyogi *et al.*, 1993; Stepanova *et al.*, 2005) and disruption of their expression is likely to reduce auxin biosynthesis in a *sur2-1gl1* background, leading to a reduction in the number of adventitious and lateral roots. Mutants of *ASA1*/*WEI2* and *ASB1*/*WEI7* had already been described to suppress mutant phenotypes of both *sur1*/*rooty1* and *sur2* mutant phenotype (Stepanova *et al.*, 2005).

Besides mutations in genes likely to be required for auxin biosynthesis, this work identified mutants in signalling and/or regulatory genes. Two allelic mutants (nos. 677, 2191) of *ABERRANT LATERAL ROOT FORMATION 4*/*ALF4* were identified (Fig. 1, Supplementary Fig. S2). *ALF4* encodes a nuclear-localized protein of unknown function that is necessary for lateral root formation (DiDonato *et al.*, 2004; Dubrovsky *et al.*, 2008). The two alleles identified had a phenotype very similar to that reported for *alf4-1* (i.e. sterile, bushy plantlets that are unable to form lateral or adventitious roots; Celenza *et al.*, 1995). Both alleles were mutated at the exact same nucleotide (Supplementary Fig. S2). This is a rare event but not impossible since it had already been shown for other mutants’ alleles (Busch *et al.*, 1996).

This study identified mutations in three genes involved in auxin signalling (Fig. 1, Supplementary Fig. S2, Supplementary Table S1). These were no. 2244, an allele of *auxin response 1* (*axr1*), no. 1626, a gain-of-function allele of *short hypocotyl 2* / *iaa3* (*shy2*), and two alleles of *rub-conjugating enzyme 1* (*rce1*) (nos. 420 and 1375.2). *AXR1* encodes a subunit of a heterodimeric RUB-activating enzyme (del Pozo *et al.*, 1998), *SHY2*/*IAA3* encodes an Aux/IAA protein (Tian and Reed, 1999), and *RCE1* is involved in the regulation of auxin signalling and ethylene biosynthesis (Dharmasiri *et al.*, 2003; Larsen and Cancel, 2004; Pacurar *et al.*, 2012). For the remaining suppressors, either no other candidate gene was clearly identified or the mapped region was still too large to choose a candidate for sequencing. With the optimization of next-generation sequencing, it will become easier to obtain a full genome sequence in order to identify the mutation.

### Phenotypic characterization of the suppressor mutants

**Fig. 3.**
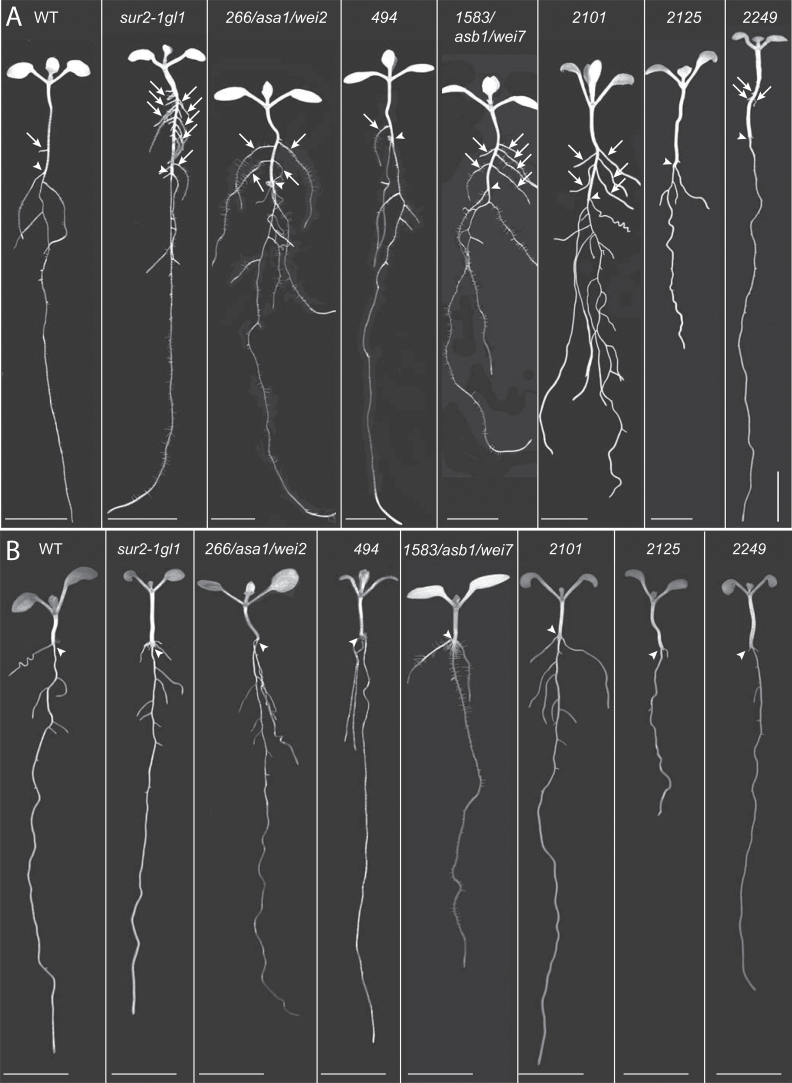
Seedling phenotypes of representative suppressor mutants. (A) Seedlings were first etiolated in the dark, until their hypocotyls were 6mm long, and then transferred to the light for 7 d. (B) Seedlings were grown in the light for 7 d. Arrowheads indicate the root–hypocotyl junction; arrows indicate adventitious roots. Bars, 5mm.

**Fig. 4.**
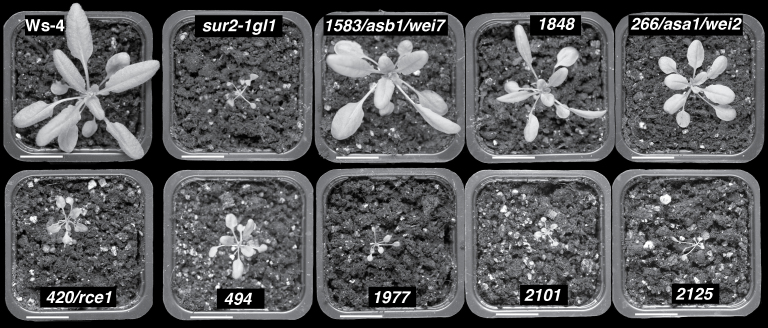
Phenotypes of 3-week-old wild-type, *sur2-1gl1*, and suppressor mutant plants. Seeds were sown *in vitro* in light conditions and were subsequently transferred to soil where the plants were grown for 3 weeks in a controlled environment, as described in Materials and methods. Bars, 20mm.

After two backcrosses with *sur2-1gl1*, a more detailed phenotypic characterization of 26 independent suppressor mutants was performed. They were characterized at the seedling level (Figs 2 and 3) and in soil (Fig. 4). *sur2-1gl1* had on average 14 AR and the wild type an average of 1.5 AR. The average number of AR for most of the suppressors lay between these two values (Fig. 2A). For a few of the mutants (nos. 1626/*shy2*, 1844, 2035, 2244/*axr1*, and 2249) the average number of AR was not significantly different from that of the wild type (Fig. 2A), and in one case (no. 2125) the average number of AR was significantly lower than in the wild type. Suppressor 2125 also had a short primary root and almost no emerged lateral roots (Figs 2B–D and 3), suggesting that the mutation alters general root development. Nevertheless, it retained some *sur2-1gl1* features such as a longer hypocotyl than the Ws-4 wild type (Figs 2E and 3B) and very small rosette with leaves bearing long petioles (Fig. 4). This suppressor is representative of suppressors that were not studied further because of their strongly altered root development.

Another type of suppressor is represented by no. 2249, which has an average number of AR closer to that of Ws-4 (Fig. 2A), but a root system closer to that of *sur2-1gl1* in terms of primary root length and lateral root density (Fig. 2B–D). No. 2249 also had a *sur2gl1-like* hypocotyl length, petioles, and epinastic cotyledons (Fig. 3A, B). Although AR and lateral roots emerged, they grew very slowly or stopped elongating (Fig. 3A, B), suggesting that no. 2249 also impacted the general root development. The mutants that are of most interest for the future are those which retain a reasonably strong root system but for which a significant reduction in the average number of AR is observed, such as nos. 1738, 1745, 1788, 1848, 1932, 1977, 2101, 2146.4, 2310, and 2344 (Figs 2A–D and 3A, B). These mutants had a hypocotyl length in between that of *sur2-1gl1* and the wild type (Fig. 2E), and their phenotype in soil varied between that of wild type and *sur2-1gl1* (Fig. 4).

### *sur2-1* suppressor mutants are not systematically reduced in free IAA content

**Fig. 5.**
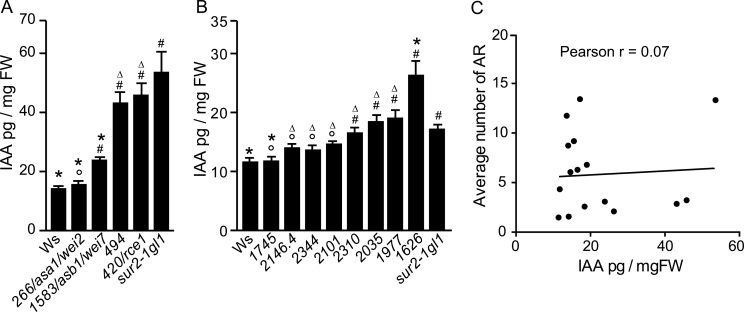
The average number of adventitious roots is not correlated with the endogenous IAA content. (A and B) Free IAA content was quantified on apical parts (cotyledons + hypocotyls) of seedlings grown *in vitro* under light for nine d; samples were extracted, purified, and analysed by LC-MRM-MS as described by Kowalczyk and Sandberg (2001) (A) or GC-MRM-MS as described by Andersen *et al.* (2008) (B). Error bars indicate standard deviation of three biological replicates; one-way ANOVA combined with the Tukey’s multiple-comparison post-test indicated that the values indicated by (*) were significantly different from *sur2-1gl1* values; hashes indicate values significantly different from wild-type Ws-4 values; ○ and Δ indicate values not significantly different from wild-type or *sur2-1gl1* values, respectively (*P*<0.05; *n*=3). (C) The average number of AR does not correlate with the endogenous IAA content; Pearson correlation coefficient *r* indicated that there is no significant correlation between the average number of AR and the endogenous IAA (*P*=0.8).

Because *sur2-1* is an auxin overproducer, this work checked the level of endogenous free IAA in the aerial part of 9-d-old seedlings of several representative suppressors grown directly in light, as described by Sorin *et al.* (2005). The measurements were performed at two different periods using Ws-4 and *sur2-1gl1* as controls (Fig. 5A, B). In the second experiment, the difference in IAA content between these two genotypes (Fig. 5B) was not as high as in the one observed in the first experiment (Fig. 5A), probably because seedlings used for Fig. 5B were not exactly at the same development stage as those used for Fig. 5A. This study group has previously shown that the endogenous content of IAA in the aerial part of *sur2-1* has a tendency to decrease after several days in the light (Barlier *et al.*, 2000). Although minimized, the difference between *sur2-1gl1* and wild type was still significant and allowed discrimination of the suppressor mutants. In the majority of the suppressors analysed, the endogenous level of auxin was not significantly different from that of the original *sur2-1gl1* line (Fig. 5). In two cases (nos. 266/*asa1*/*wei2* and 1745), the free IAA content was similar to wild-type level (Fig. 5A, B) suggesting that the corresponding mutations could alter expression of genes involved in or controlling IAA biosynthesis. As stated above, no. 266 is allelic to *asa1*/*wei2* and is likely to be affected in tryptophan, and thus IAA, biosynthesis. Mutant 1745 remains to be identified, and since no obvious candidate gene is present in the mapped region it is expected that a new player in the regulation of auxin metabolism will be identified. For no. 1626/*shy2*, the auxin content was higher than in *sur2-1gl1* (Fig. 5B). No. 1626 is a dominant mutation affecting expression of the *SHY2*/*IAA3* gene (Tian and Reed, 1999), which acts as a negative regulator of auxin signalling (Tian *et al.*, 2002); therefore, the increased endogenous auxin level is likely to be explained by a feedback regulation due to the fact that the mutant cannot sense auxin. Previous work has shown that AR development in *Arabidopsis* hypocotyl does not necessarily correlate with the endogenous auxin content (Sorin *et al.*, 2006; Gutierrez *et al.*, 2012). The current work shows that *sur2-1* suppressor mutants retaining wild-type level of IAA (nos. 266/*asa1*/*wei2* and 1745) still produce more AR than the wild type, whereas others with an IAA level similar to *sur2-gl1* (e.g. nos. 420/*rce1*, 494, 1977, and 2035) produce significantly fewer AR than *sur2-1gl1.* These results corroborate the fact that the number of AR developed in the *Arabidopsis* hypocotyl is not necessarily correlated to the endogenous content of auxin (Fig. 5C).

### A role for ethylene in adventitious root initiation in *Arabidopsis* hypocotyl

**Fig. 6.**
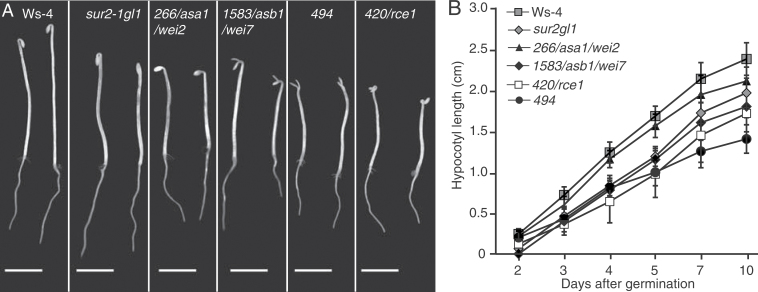
Ethylene-related phenotypes of selected suppressor mutants. (A) Seedlings were grown in the dark for 5 d; nos. 266/*asa1*/*wei2*, 420/*rce1*, and 1583 show a reduced apical hook compared to wild type or *sur2-1gl1*, whereas no. 420/*rce1* has an increased apical hook; arrowheads indicate the root–hypocotyl junction; bars, 5mm. (B) Hypocotyl length of wild type, *sur2-1gl1*, and suppressors 266/*asa1*/*wei2*, 420/*rce1*, 494, and 1583 grown *in vitro* in the dark; the hypocotyl was measured at different time points; error bars indicate standard error.

Three of the genes identified are involved in auxin biosynthesis (nos. 266/*ASA1*/*WEI2* and 1583/*ASB1*/*WEI7*; Stepanova *et al.*, 2005) or auxin signalling (no. 420/*RCE1*; del Pozo and Estelle, 1999; Dharmasiri *et al.*, 2003). These are also linked to ethylene signalling or biosynthesis since the *wei2* and *wei7* mutants have been described as weak ethylene-insensitive mutants (Stepanova *et al.*, 2005) and it was demonstrated that the *RCE1* gene is required for a proper regulation of ethylene biosynthesis (Larsen and Cancel, 2004). The *rce1-2* allele was shown to overproduce ethylene and displayed the characteristic ethylene-related triple-response phenotype when grown in the dark (Larsen and Cancel, 2004). Because ethylene was shown to play a role in the regulation of AR formation in other species (da Costa *et al.*, 2013), these suppressors were characterized further. When grown in the dark, seedlings of nos. 266/*asa1*/*wei2*, 1583/*asb1*/*wei7*, and 420/*rce1* showed a shorter hypocotyl than the wild type (Fig. 6A, B) and a reduced apical hook for nos. 266/*asa1*/*wei2* and 1583/*asb1*/*wei7* or an accentuated apical hook in the case of no. 420/*rce1* (Fig. 6A; Pacurar *et al.*, 2012). Indeed, these are the characteristic phenotypes of *asa1*/*wei2*, *asb1*/*wei7*, and *rce1* mutants. The suppressor mutant *494* had a phenotype similar to nos. 266/*asa1*/*wei2* or 1583/*asb1*/*wei7* when grown in the dark but had no apical hook 5 d after germination (Fig. 6), suggesting that it could also be affected in ethylene response or biosynthesis.

**Fig. 7.**
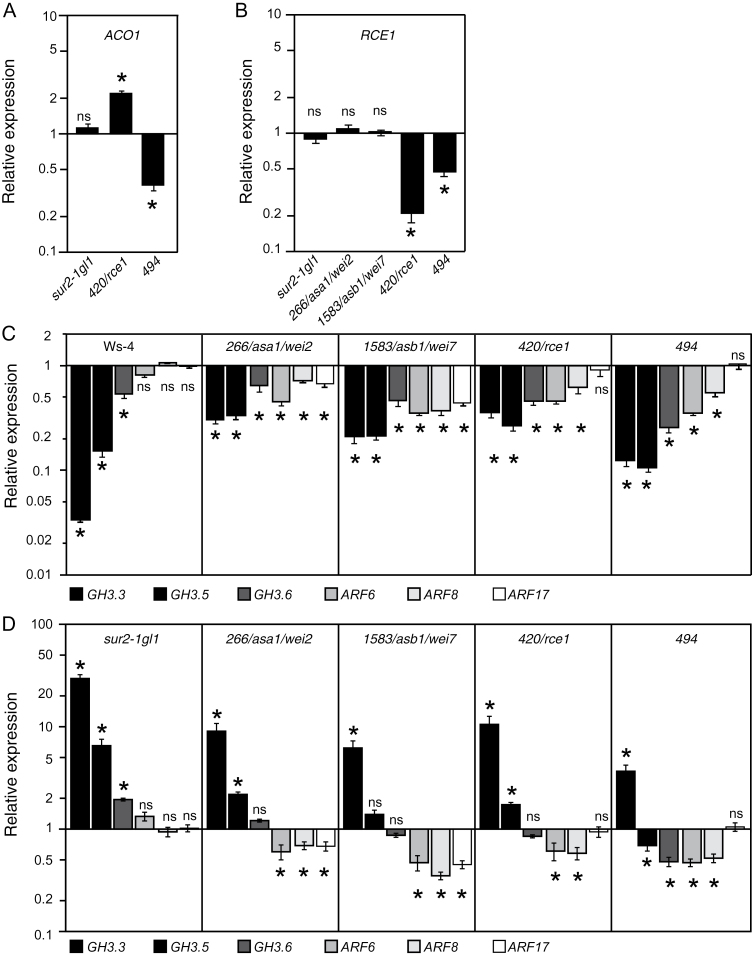
Impact of suppressor mutation on the ARF/GH3 regulatory module. (A) Relative transcript amounts of *ACO1* in wild type (Ws-4), *sur2-1gl1*, and suppressors 420/*rce1* and 494. (B) Relative *RCE1* transcript amounts in wild type (Ws-4), *sur2-1gl1*, and suppressors 266/*asa1*/*wei2*, 1583/*asb1*/*wei7*, 420/*rce1*, and 494. (C and D) Quantification by quantitative real-time PCR of *GH3.3*, *GH3.5*, *GH3.6*, *ARF6*, *ARF8*, and *ARF17* transcript abundance in hypocotyls of seedlings (wild type Ws-4, *sur2-1gl1*, and suppressors 266/*asa1*/*wei2*, 420/*rce1*, 494, and 1583/*asb1*/*wei7*), which were etiolated until their hypocotyl had reached 6mm and then transferred to the light for 72h. (A, B, and D) Gene expression values are relative to the expression in the wild type, for which the value is set to 1. (C) Gene expression values are relative to the expression in *sur2-1gl1*, for which the value is set to 1. Error bars indicate standard error obtained from three independent biological replicates; ns indicates values not significantly different from wild-type (A, B, D) or *sur2-1gl1* (C) values by one-way ANOVA combined with the Dunnett’s comparison post-test; asterisks indicate values that were significantly different (*P*<0.05; *n*=3).

To discriminate between potential ethylene insensitivity or modification of ethylene biosynthesis, this work quantified the expression of the *ACO* gene, which encodes the 1-aminoacyclopropane-1-carboxylic acid (ACC) oxidase. It has previously been shown that ethylene biosynthesis was correlated with ACO activity (Abeles *et al.*, 1992; Larsen and Cancel, 2004) and it was anticipated that overexpression or downregulation of *ACO1* would result in an up- or downregulation of ethylene production. The relative transcript abundance of *ACO1* was unchanged in the *sur2-1gl1* mutant compared to the wild-type level (Fig. 7A), whereas it was significantly increased in no. 420/*rce1* and significantly reduced in no. 494. This coincides with the wild-type-like apical hook phenotype of *sur2-1gl1* and the increased or reduced apical hook of nos. 420/*rce1* and 494, respectively. Because ethylene was shown to promote AR development in rice and tomato (Clark *et al.*, 1999)m a decrease in ethylene biosynthesis could explain the reduced number of AR in no. 494. However, no. 420/*rce1,* which in contrast is likely to overproduce ethylene, when compared to no. 494, has a similar reduction in average AR number. In addition, it is interesting to note that the relative abundance of the *RCE1* transcript is unchanged in *sur2-1gl1* and nos. 266/*asa1*/*wei2* and 1583/*asb1*/*wei7* mutants compared to wild-type level, but is significantly reduced in no. 494 (Fig. 7B). This was unexpected, considering that no. 494 had an opposite ethylene-related apical hook phenotype compared to no. 420/*rce1.* Nevertheless, it has been reported that ethylene could have differential effect on adventitious rooting, depending on the stage of the induction phase (da Costa *et al.*, 2013). Therefore, although it seems unlikely that the phenotypes of nos. 420/*rce1* and 494 mutants are due to modification of endogenous ethylene level, the current work cannot exclude that, in no. 494, a reduced ethylene production has a negative effect on the early events of AR induction phase, whereas an excess of ethylene in no. 420/*rce1* could inhibit a later stage during the induction phase. Future identification of the no. 494 mutation and further characterization of these suppressor mutations both in the *sur2-1gl1* and in the wild-type background could help understanding of how ethylene and auxin interact in the control of adventitious rooting.

### *sur2-1* suppressor mutations differentially affect the ARF/GH3 regulatory module

In the past few years, studies have shown that a regulatory module, composed of three *AUXIN RESPONSE FACTOR* (*ARF*) genes (*ARF6*, *ARF8*, and *ARF17*) and three auxin-responsive genes (*GH3.3*, *GH3.5*, and *GH3.6*), controls adventitious rooting in *Arabidopsis* hypocotyls (Gutierrez *et al*., 2009, 2012). ARF proteins bind AREs present in the promoters of auxin-inducible genes such as *Aux*/*IAA*, *SAUR*, and *GH3* and either repress or activate their transcription (Takase *et al.*, 2003; Tiwari *et al.*, 2003). Whereas ARF6 and ARF8 positively regulate *GH3.3*, *3.5*, and *3.6* expression and thereby adventitious rooting, ARF17 is a negative regulator. The average number of AR positively correlates with the abundance of *GH3.3*, *3.5*, and *3.6* transcripts and proteins (Sorin *et al.*, 2006; Gutierrez *et al.*, 2012). Thus, the current work checked whether nos. 266/*ASA1*/*WEI2*, 420/*RCE1*, 1583/*ASB1*/*WEI7*, and 494 mutations had an impact on the ARF/GH3 regulatory module. The relative transcript amount of the three *ARF* and three *GH3* genes were quantified in these suppressor mutants and compared to *sur2-1gl1* (Fig. 7C) and wild-type levels (Fig. 7D). In *sur2-1gl1*, the relative transcript amounts of the three *GH3* genes were significantly higher than in the wild type, although those of *ARF6*, *8*, and *17* were unchanged (Fig. 7D). This suggests that the induction by auxin of the expression of the three *GH3* genes is not due to a modification of the *ARF* transcript level.

ARFs proteins have a conserved N-terminal DNA-binding domain that binds to consensus AREs present in the promoter of auxin-inducible genes. In most cases, ARFs also contain a conserved C-terminal dimerization domain required for the heterodimerization with Aux/IAA proteins that repress the transcriptional activity of ARF proteins. The presence of auxin triggers the degradation, via the 26S ubiquitine-proteasome pathway, of Aux/IAA transcriptional repressors (reviewed in Chapman and Estelle, 2009). This facilitates the expression of auxin-inducible genes such as *GH3* genes. Since *ARF6*, *ARF8*, and *ARF17* transcript levels are not affected in *sur2-1gl1*, the overexpression of *GH3* genes is likely due to the auxin-induced degradation of the Aux/IAA partners of ARF6 and ARF8.

**Fig. 8.**
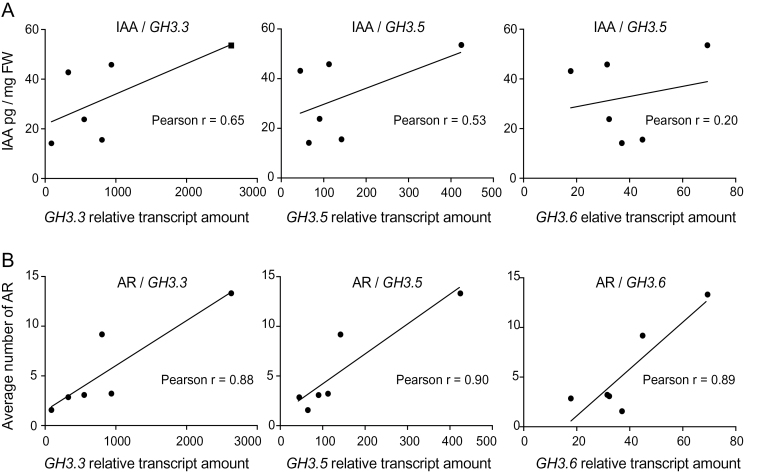
Correlation analysis of *GH3.3*, *GH3.5*, and *GH3.6* relative transcript amounts with the endogenous IAA content or the number of adventitious roots. (A) *GH3.3*, *GH3.5*, and *GH3.6* relative transcript amounts are not correlated with the endogenous level of IAA; Pearson correlation coefficient *r* indicated that there was no significant correlation between the endogenous IAA content in the hypocotyl and the relative amount of *GH3* transcripts; *P*=0.16, 0.27, and 0.69 for *GH3.3*, *GH3.5*, and *GH3.6*, respectively. (B) *GH3.3*, *GH3.5*, and *GH3.6* relative transcript amounts are positively correlated with the average number of adventitious roots; Pearson correlation coefficient *r* indicated that there was a significant positive correlation between the average number of AR and the relative amount of *GH3* transcripts; *P*=0.02, 0.01, and 0.02 for *GH3.3*, *GH3.5*, and *GH3.6,* respectively. *GH3* gene expression values are relative to the expression level of *APT1*, which was used as a reference gene, as described in Materials and methods.

In the four suppressors analysed, the reduced number of AR coincided with a significant decrease of the three *GH3* genes compared to *sur2-1gl1* (Fig. 7C). In nos. 266/*asa1*/*wei2* and 1583/*asb1*/*wei7*, this could be explained by the reduction of the endogenous auxin content in the hypocotyl (Fig. 5A), but this is unlikely to be the only explanation. Indeed the relative transcript amount of the *GH3* genes in nos. 266/*asa1*/*wei2* and 1583, although significantly lower than in *sur2-gl1*, was still higher than in the wild type (Fig. 7D), which may explain the fact that these two mutants still have more AR than the wild type. In addition, in nos. 420/*rce1* and 494 mutants, the relative expression of the *GH3* genes is much lower than in *sur2-1gl1*, despite the endogenous auxin level remaining unchanged compared to *sur2-1gl1* (Fig. 5A). In fact, the *GH3.3*, *GH3.5*, and *GH3.6* relative transcript amounts in the suppressor mutants analysed did not correlate with the endogenous auxin content (Fig. 8A) but positively correlated with the number of adventitious roots (Fig. 8B). Since the endogenous IAA content cannot explain the reduction of the relative amount of *GH3* transcripts in the suppressor mutants, this reduction is likely due to the downregulation of the regulatory *ARF* expression. In nos. 266/*asa1*/*wei2* and 1583/*asb1*/*wei7*, both positive regulators (*ARF6*, *ARF8)*, and the negative regulator (*ARF17*) are downregulated, whereas in nos. 420/*rce1* and 494, the relative transcript amount of *ARF17* remains unchanged (Fig. 7C, D). In these latter cases, the balance of transcriptional regulators is strongly in favour of the repressor, which according to previous data (Gutierrez *et al.*, 2012) is likely repressing *GH3* gene expression despite the elevated level of auxin.

The initial phenotypic characterization of these four suppressors suggests that the decreased number of AR could be due to perturbation of ethylene–auxin crosstalk, interpretation also supported by the recently shown coregulation of both ethylene and auxin biosynthesis by the enzyme VAS1 encoding pyridoxal-phosphate-dependent aminotransferase (Zheng *et al.*, 2013). However, characterization of the expression of genes involved the core *ARF*/*GH3* regulatory module shows that modification of the endogenous levels of auxin or ethylene is not directly responsible for the suppressor phenotypes. Besides the AR phenotype, the common feature between these four suppressors is the modification of the balance between the three *ARF* and three *GH3* genes. The future identification of the gene mutated in no. 494 is likely to provide more clues on the molecular interactions involved in this pathway.

### Concluding remarks

From numerous studies addressing different aspects of AR formation in a variety of species, it is obvious that adventitious rooting is controlled by many genes and exhibits considerable phenotypic plasticity. Genomics, transcriptomics, and proteomics studies performed with several species have started to unveil its complex genomic control and interactions (Kohler *et al.*, 2003; Brinker *et al.*, 2004; Sorin *et al.*, 2006; Ramirez-Carvajal *et al.*, 2009; Muthreich *et al.*, 2013; Thirunavukkarasu *et al.*, 2013; Wei *et al.*, 2013). The current contribution performed a screen to identify new players involved in AR formation in the model plant *A. thaliana*. The findings confirm the multitude and variety of genetic controls of AR formation by identifying to date suppressor genes involved in auxin biosynthesis (*ASA1*/*WEI2*, *ASB1*/*WEI7*, *TSB1*), auxin signalling (*SHY2*, *AXR1*, *RCE1*), and lateral root formation (*ALF4*), which can also regulate AR formation. Numerous suppressor mutants remain to be identified and since no obvious candidate gene is present in the mapped regions, this study group expects to identify new regulators of the intricate network controlling AR formation. These are likely to act at the crosstalk of auxin and other signalling pathways.

## Supplementary material

Supplementary data are available at *JXB* online.

Supplementary Fig. S1. Outline of the screening procedure and the induction of AR on the etiolated *Arabidopsis* hypocotyls.

Supplementary Fig. S2. The structure of the identified suppressor genes.

Supplementary Table S1. Segregation analysis of *sur2* suppressor mutants.

Supplementary Table S2. Sequences of primers used for quantifying target genes by quantitative real-time PCR.

Supplementary Data
