# Supplementary material for: Identification of new adventitious rooting mutants amongst suppressors of the Arabidopsis thaliana superroot2 mutation
Source: J Exp Bot. 2014 Mar 4;65(6):1605–18. doi: 10.1093/jxb/eru026 (PMC3967091; doi:10.1093/jxb/eru026)
Supplement: Supplementary Data [file supp_65_6_1605__index.html]

Identification of new adventitious rooting mutants amongst suppressors of the Arabidopsis thaliana superroot2 mutation — Identification of new adventitious rooting mutants amongst suppressors of the Arabidopsis thaliana superroot2 mutation — Supplementary Data 

# Identification of new adventitious rooting mutants amongst suppressors of the *Arabidopsis thaliana superroot2* mutation

## Supplementary Data

Data files

**Files in this Data Supplement:**

- Supplementary Data - Supplementary Data
