# Supplementary material for: Identification of new adventitious rooting mutants amongst suppressors of the Arabidopsis thaliana superroot2 mutation
Source: J Exp Bot. 2014 Mar 4;65(6):1605–18. doi: 10.1093/jxb/eru026 (PMC3967091; doi:10.1093/jxb/eru026)
Supplement: Supplementary Data [file supp_eru026_jexbot112474_file001.pdf]

Journal of Experimental Botany

Screening for suppressors of the *superroot2* mutation in *Arabidopsis thaliana* identifies new adventitious rooting mutants

Daniel Ioan Pacurar, Monica Lacramioara Pacurar, John Desmond Bussell, Joseli Schwambach, Tiberia Ioana Pop, Mariusz Kowalczyk, Laurent Gutierrez, Emilie Cavel, Salma Chaabouni, Karin Ljung, Arthur Germano Fett-Neto, Doru Pamfil and Catherine Bellini

Supplementary Fig. S1. Outline of the screening procedure (A), and the induction of AR on the etiolated Arabidopsis hypocotyls (B)

Figure S1

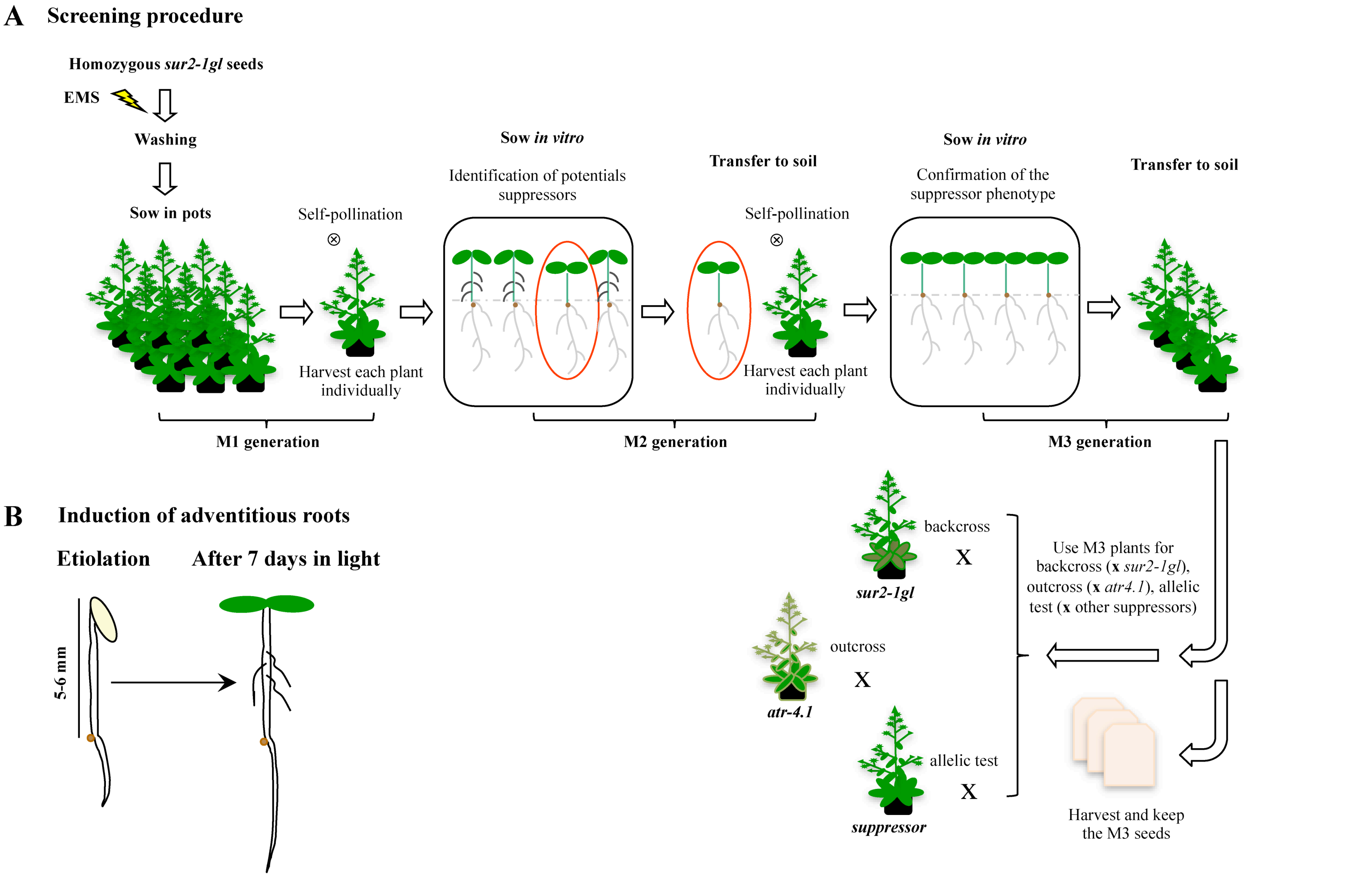

Supplementary Fig. S2. The structure of the identified suppressor genes

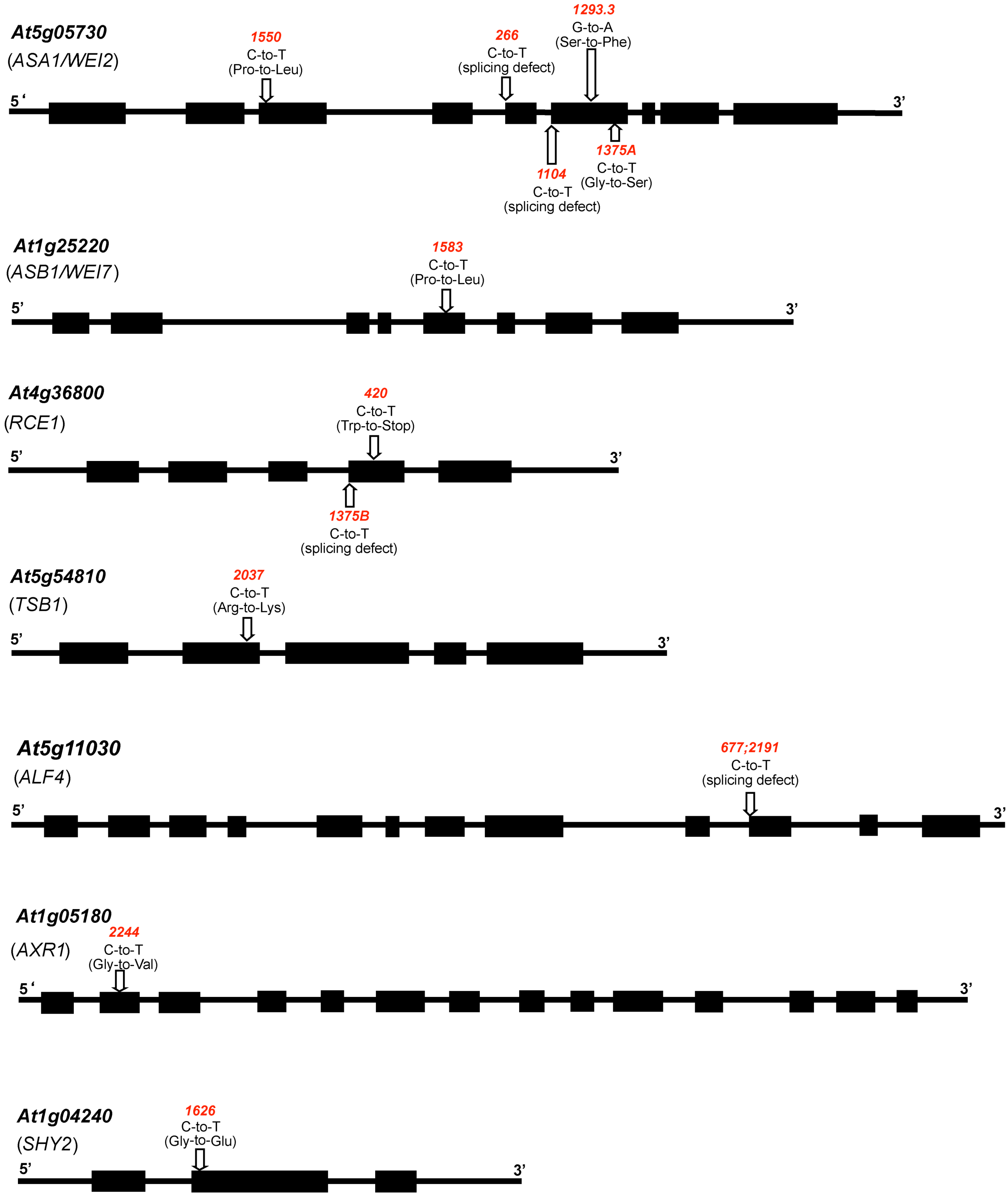

Exons are represented by boxes, lines between them represent the introns. The arrows indicate the position of the point mutations in the corresponding suppressors, shown in red. The type and the nature of mutations as compared to wild type are indicated in brackets.

Supplementary Table S1. Segregation analysis of *sur2* suppressor mutants

Suppressor line number (*Gene name*)	N° seeds sown	N° of *sur2-1gl1* observed	N° of suppressors observed	N° of *sur2-1gl1* expected for 3/1 segregation	N° of suppressors expected for 3/1 segregation	χ^2	significance
266	105	89	16	78.75	26.25	5.34	s
420 (*RCE1)*	124	94	30	106.5	35.5	0.39	ns
420.3 (*ASA1/WEI2*)	NA	NA	NA	NA	NA	NA	NA
494	111	85	26	83.25	27.75	0.15	ns
677 (*ALF4*)	100	83	17	75	25	3.41	ns
1104 (*ASA1/WEI2*)	NA	NA	NA	NA	NA	NA	NA
1293.2	161	127	34	120.75	40.25	1.29	ns
1293.3 (*ASA1/WEI2*)	104	80	24	78	26	0.7	ns
1319.1	132	110	22	99	33	4.89	s
1319.2 (*ASA1/WEI2*)	NA	NA	NA	NA	NA	NA	NA
1375.1 (*ASA1/WEI2*)	NA	NA	NA	NA	NA	NA	NA
1375.2 (*RCE1*)	NA	NA	NA	NA	NA	NA	NA
1550 (*ASA1/WEI2*)	81	67	14	60.75	20.25	2.57	ns
1583 (*ASB1/WEI7*)	137	105	32	102.75	34.25	0.7	ns
1623	NA	NA	NA	NA	NA	NA	NA
1626 (*IAA3/SHY2*)	148	33	115	37	111	0.58	ns
1738	143	101	42	107.25	35.75	1.46	ns
1745	223	175	48	167.25	55.75	1.44	ns
1747	192	148	44	144	48	0.44	ns
1759	228	185	43	171	57	4.58	s
1788	158	116	42	118.5	39.5	0.21	ns
1806	114	97	17	85.5	28.5	6.19	s
1844	131	116	15	98.25	32.75	12.83	s
1848	294	218	76	220.5	73.5	0.11	ns
1835 (*ASB1/WEI7*)	NA	NA	NA	NA	NA	NA	NA
1863	141	106	35	105.75	35.25	0.00	ns
1888	146	117	29	109.5	36.5	2.05	ns

Supplementary Table S1 continued. Segregation analysis of *sur2* suppressor mutants

Suppressor line number	N° seeds sown	N° of *sur2-1gl1* observed	N° of suppressors observed	N° of *sur2-1gl1* expected for 3/1 segregation	N° of suppressors expected for 3/1 segregation	χ^2	significance
1922 (*ASA1/WEI2*)	NA	NA	NA	NA	NA	NA	NA
1932	102	78	24	76.5	25.5	0.12	ns
1977	354	319	35	265.5	88.5	43.12	s
2032	240	183	47	180	60	2.87	ns
2035	145	111	34	108.75	36.25	0.19	ns
2037	NA	NA	NA	NA	NA	NA	NA
2041 (*TSB1)*	NA	NA	NA	NA	NA	NA	NA
2059	NA	NA	NA	NA	NA	NA	NA
2101	241	210	31	180.75	60.25	18.93	s
2125	144	111	33	108	36	0.33	ns
2146.1	146	131	15	109.5	36.5	16.89	s
2146.4	242	199	43	181.5	60.5	6.75	s
2244 (*AXR1*)	153	115	38	114.75	38.25	0.00	ns
2249	191	147	44	143.25	47.75	0.4	ns
2191 (*ALF4*)	NA	NA	NA	NA	NA	NA	NA
2293 (*TSB1*)	NA	NA	NA	NA	NA	NA	NA
2307 (*TSB1*)	149	139	10	111.75	37.25	26.58	s
2310	139	111	28	104.25	34.75	1.75	ns
2344	212	171	41	159	53	3.62	ns

In the suppressor column, the corresponding mutated gene when identified is indicated in brackets.
NA: not analyzed
$\chi 2$: Expected values were based on an the assumption of segregation at a single recessive locus yielding a ratio of 3:1 for *sur2-1gl1* to suppressor mutant seedlings.
s = significant, ns = not significant ($P < 0.05$). Note that suppressor 1626 is a dominant mutation, and for this line the expected ratio was instead 1:3 *sur2-1gl1* to suppressor mutant seedlings.

Supplementary Table S2. Sequences of primers used for quantifying target genes by quantitative RT-PCR

Amplicon name	Gene	Forward primer	Reverse primer
ACO1	At2g19590	GCGGAGTTGTGGATTACGTT	TTCCAAAGCCAAACTGAAGC
APT1	At1g27450	GAGACATTTTGCGTGGGATT	CGGGGATTTTAAGTGGAACA
ARF6 uncleaved	At1g30330	CAAAGTTTAGCAGCTACCACGA	ACGTCGTTCTCTCGGTCAAC
ARF8 uncleaved	At5g37020	TTTGCTATCGAAGGGTTGTTG	CATGGGTCATCACCAAGGA
ARF17 uncleaved	At1g77850	GCACCTGATCCAAGTCCTTC	GGTGAATAGCTGGGGAGGAT
GH3.3	At2g23170	ACAATTCCGCTCCACAGTTC	ACGAGTTCCTTGCTCTCCAA
GH3.5	At4g27260	GTCTTCGAGGACTGCTGCTT	ATGTCCCTGGCTCAACAATC
GH3.6	At5g54510	CCTTGTTCCGTTTGATGCTT	CGTGTTACCGTTCAAGCAGA
RCE1	At4g36800	TGTCGGTCAGACCTTTTTCC	CAAACGAGGGTCCTTGAGAA
TIP41	At4g34270	GCTCATCGGTACGCTCTTTT	TCCATCAGTCAGAGGCTTCC
